# Lipoprotein(a) Does Not Predict Thrombotic Events and In-Hospital Outcomes in Patients with COVID-19

**DOI:** 10.3390/jcm12103543

**Published:** 2023-05-18

**Authors:** Vanessa Bianconi, Massimo R. Mannarino, Federica Ramondino, Jessica Fusaro, Francesco Giglioni, Marco Braca, Federica Ricciutelli, Rita Lombardini, Rita Paltriccia, Alessia Greco, Iliana C. Lega, Matteo Pirro

**Affiliations:** 1 Unit of Internal Medicine, Department of Medicine and Surgery, University of Perugia, 06129 Perugia, Italy; federica.ramondino@studenti.unipg.it (F.R.); jessica.fusaro@studenti.unipg.it (J.F.); francesco.giglioni@studenti.unipg.it (F.G.); marco.braca@studenti.unipg.it (M.B.); federica.ricciutelli@studenti.unipg.it (F.R.); rita.lombardini@unipg.it (R.L.); rita.paltriccia@unipg.it (R.P.); alessia.greco@unipg.it (A.G.); matteo.pirro@unipg.it (M.P.); 2 Women’s College Research Institute, Women’s College Hospital, Toronto, ON M5G 1N8, Canada; massimo.mannarino@unipg.it (M.R.M.); iliana.lega@wchospital.ca (I.C.L.)

**Keywords:** COVID-19, CRP, D-dimer, inflammation, Lp(a), procalcitonin, SARS-CoV-2, thrombosis, WBC

## Abstract

The prothrombotic and proinflammatory properties of lipoprotein(a) (Lp(a)) have been hypothesized to play a role in the pathogenesis of severe COVID-19; however, the prognostic impact of Lp(a) on the clinical course of COVID-19 remains controversial. This study aimed to investigate whether Lp(a) may be associated with biomarkers of thrombo-inflammation and the occurrence of thrombotic events or adverse clinical outcomes in patients hospitalized for COVID-19. We consecutively enrolled a cohort of patients hospitalized for COVID-19 and collected blood samples for Lp(a) assessment at hospital admission. A prothrombotic state was evaluated through D-dimer levels, whereas a proinflammatory state was evaluated through C-reactive protein (CRP), procalcitonin, and white blood cell (WBC) levels. Thrombotic events were marked by the diagnosis of deep or superficial vein thrombosis (DVT or SVT), pulmonary embolism (PE), stroke, transient ischemic attack (TIA), acute coronary syndrome (ACS), and critical limb ischemia (CLI). The composite clinical end point of intensive care unit (ICU) admission/in-hospital death was used to evaluate adverse clinical outcomes. Among 564 patients (290 (51%) men, mean age of 74 ± 17 years) the median Lp(a) value at hospital admission was 13 (10–27) mg/dL. During hospitalization, 64 (11%) patients were diagnosed with at least one thrombotic event and 83 (15%) patients met the composite clinical end point. Lp(a), as either a continuous or categorical variable, was not associated with D-dimer, CRP, procalcitonin, and WBC levels (*p* > 0.05 for all correlation analyses). In addition, Lp(a) was not associated with a risk of thrombotic events (*p* > 0.05 for multi-adjusted odds ratios) nor with a risk of adverse clinical outcomes (*p* > 0.05 for multi-adjusted hazard ratios). In conclusion, Lp(a) does not influence biomarkers of plasma thrombotic activity and systemic inflammation nor has any impact on thrombotic events and adverse clinical outcomes in patients hospitalized for COVID-19.

## 1. Introduction

The coronavirus disease 2019 (COVID-19) pandemic spread worldwide, leading to hundreds of millions of diagnosed cases and over 6.5 million deaths as of March 2023 [1]. Although COVID-19-related mortality has been drastically reduced with the availability of anti-SARS-CoV-2 vaccines, severe forms of COVID-19 continue to be a threat for healthcare systems due to persistently high hospitalization and mortality rates, especially in geographical areas where vaccination coverage is lower [2,3,4,5]. Hence, the search for novel prognostic markers and therapeutic targets involved in the pathogenesis of COVID-19 continues to be of relevance [6,7,8,9,10].

So-called thrombo-inflammation, that is, the pathogenic interaction between thrombosis and systemic inflammation, has been widely recognized as a crucial step in COVID-19 progression towards its most severe and life-threatening clinical manifestations [11,12,13,14,15]. Accordingly, the detection of laboratory and instrumental indices of thrombosis, as well as the measurement of circulating levels of different inflammatory biomarkers, are routinely used to guide the clinical management of patients hospitalized for COVID-19 [16,17,18]. In addition, both antithrombotic and anti-inflammatory therapies are the cornerstone of COVID-19 therapeutic management [19,20,21].

Given its putative prothrombotic and proinflammatory properties, lipoprotein(a) (Lp(a)), a low-density lipoprotein (LDL) particle carrying an apolipoprotein(a) moiety covalently bound to apolipoprotein B, has been hypothesized to be a possible driver of a worse clinical course among patients with COVID-19 [22,23]. Indeed, due to its content in proinflammatory oxidized phospholipids, Lp(a) might directly promote endothelium injury and the impairment of endothelial antithrombotic activity [22,23,24,25]. Furthermore, Lp(a) might directly act as an antifibrinolytic factor, promoting thrombosis progression due to its structure similarity with plasminogen [22,23,24,25]. Although circulating Lp(a) is mostly genetically determined, an increase in circulating Lp(a) levels may occur in different inflammatory conditions due to the activation of the interleukin-6 (IL-6) response element on the LPA gene codifying for apolipoprotein(a) [22,23]. Thus, the amplification of the thrombo-inflammation vicious circle may occur in patients with COVID-19 and elevated Lp(a) levels, which may ultimately impair clinical prognoses.

Only a few studies have investigated the association between circulating Lp(a) levels and different biomarkers of systemic inflammation and plasma thrombotic activity in the context of COVID-19, showing inconsistent results [26,27,28,29]. Additionally, data on the association between Lp(a) and either COVID-19 thrombotic complications or COVID-19 in-hospital outcomes are limited and contrasting [26,28,29,30,31].

This study aimed to investigate whether Lp(a) may be associated with biomarkers of thrombo-inflammation and the occurrence of thrombotic events, either arterial or venous, or adverse clinical outcomes in a cohort of patients hospitalized for COVID-19.

## 2. Materials and Methods

### 2.1. Enrollment of the Study Population

A cohort of patients admitted for COVID-19 to the internal medicine ward of the “Santa Maria della Misericordia” hospital of Perugia (Italy) from February 2021 to October 2022 was consecutively enrolled. The study protocol was developed in accordance with the principles of the Helsinki Declaration and approved by the local ethics committee. The inclusion criteria were as follows: (1) age ≥ 18 years; (2) a positive result on a real-time reverse transcriptase PCR (RT-PCR) assay testing for SARS-CoV-2 on nasal or pharyngeal swab specimens at hospital admission; and (3) written informed consent within 24 h of hospital admission. Previous lower-limb amputation was an exclusion criterion. At enrollment all patients underwent blood sampling for the assessment of Lp(a), biomarkers of thrombo-inflammation (i.e., D-dimer, C-reactive protein (CRP), procalcitonin, and white blood cell (WBC) count), and other routine laboratory parameters. In addition, all patients were screened for deep vein thrombosis (DVT) or superficial vein thrombosis (SVT) via venous Doppler ultrasound of the lower limbs. Afterwards, the overall clinical management followed currently available scientific recommendations and routine medical practice.

### 2.2. Baseline Data Collection

Data on demographic characteristics, coexisting medical conditions, current treatments, laboratory tests, and physical examinations performed at hospital admission were collected and registered in medical records. Standard laboratory techniques were used to determine the following laboratory parameters: blood gas parameters (ABL90 FLEX blood gas analyzer, Radiometer, Copenhagen, Denmark), procalcitonin (VIDAS PCT assay, bioMérieux, Lyon, France), platelet and WBC count (Sysmex XT-2000i, Dasit, Cornaredo, Italy), D-dimer (BCS XP Coagulation Analyzer, Siemens, Munich, Germany), CRP, creatinine, bilirubin (AU5800 Clinical Chemistry System, Beckman Coulter, Brea, CA, USA), total cholesterol, high-density lipoprotein (HDL) cholesterol, and triglycerides (KONE-PRO Autoanalyzer, Dasit). Lp(a) was measured via nephelometry through an apo(a) isoform-dependent assay (BN-II-System, Siemens HD). The Friedewald formula was used to calculate low-density lipoprotein (LDL) cholesterol [32]. The following clinical parameters were recorded among those obtained during physical examination at hospital admission: body mass index (BMI), respiratory rate, oxygen saturation (SpO_2_), fraction of inspired oxygen (FiO_2_), Glasgow Coma Scale (GCS), systolic blood pressure (SBP), and diastolic blood pressure (DBP) [33,34]. The severity of COVID-19 at hospital admission was established according to the National Institute of Health (NIH) classification [20]. The Charlson comorbidity index (CCI) was calculated for each patient by integrating information on coexisting medical conditions [35,36]. The sequential organ failure assessment (SOFA) score was estimated for each patient based on clinical and laboratory data at admission [36,37]. The Padua prediction (PP) score was evaluated for each patient to assess the risk of venous thromboembolism (VTE) at the study baseline [38].

### 2.3. Clinical End Points

All patients were followed up until the occurrence of the composite end point of ICU admission/in-hospital death or hospital discharge. Data on therapeutic management and the occurrence of selected clinical end points (i.e., thrombotic events, ICU admission, and in-hospital death) were registered in medical records. Thrombotic events were categorized as venous thrombotic events (DVT or SVT, either screened at hospital admission or diagnosed in symptomatic patients during the hospital stay via venous Doppler ultrasound of the lower limbs; pulmonary embolism (PE), detected by pulmonary angiography in symptomatic patients) or arterial thrombotic events (transient ischemic attack (TIA) and stroke, identified by observing the onset of new focal neurological signs/symptoms and confirmed with magnetic resonance imaging (MRI) or computed tomography (CT) imaging; acute coronary syndrome (ACS), diagnosed in symptomatic patients on the basis of electrocardiographic/echocardiographic signs associated with an elevation in troponin; and critical limb ischemia (CLI), detected in symptomatic patients via arterial Doppler ultrasound of the lower limbs).

### 2.4. Statistical Analysis

The SPSS statistical package, release 24.0 (SPSS Inc., Chicago, IL, USA), was used for all statistical analyses. The Shapiro test was used to verify the normality of the study variables. Categorical variables were expressed as percentages, while continuous variables were expressed as the mean ± standard deviation (SD) or median (interquartile range (IQR)). Correlation analyses were performed via the use of Spearman’s coefficient of correlation. The independent samples *t*-test, the Mann–Whitney U-test, and the chi-squared test were used for two-group comparisons, whereas the Kruskal–Wallis test and the chi-squared test were used for multiple-group comparisons. Unadjusted and multi-adjusted logistic regression analyses were performed to assess the association between Lp(a) and thrombotic events, either arterial and venous combined or separated. Unadjusted and multi-adjusted Cox regression analyses were used to explore the association between Lp(a) and the composite end point of ICU admission/in-hospital death. In multi-adjusted analyses, demographic variables and clinical variables differing significantly according to the selected end points were included as potential confounders, along with anti-SARS-CoV-2 vaccines. Explorative logistic regression and Cox regression analyses, either unadjusted or multi-adjusted, were also performed to explore the association between Lp(a) and the risk of thrombotic events and adverse clinical outcomes in the subgroup of patients with severe COVID-19. For all analyses, Lp(a) was managed either as a continuous variable or as a categorical variable (i.e., Lp(a) ≤ versus > the median value, Lp(a) ≤ versus > 30 mg/dL, Lp(a) ≤ versus > 50 mg/dL, or Lp(a) tertiles). The median value of Lp(a) was arbitrarily chosen as a possible threshold with which to discriminate between low and high Lp(a) in the study population. The Lp(a) values of 30 mg/dL and 50 mg/dL were chosen as possible thresholds with which to discriminate high and low Lp(a) based on the last European Atherosclerosis Society consensus statement, suggesting their value to rule out and rule in a significant increase in CV risk, respectively [39].

## 3. Results

### 3.1. Characteristics of the Study Population

**Figure 1:**
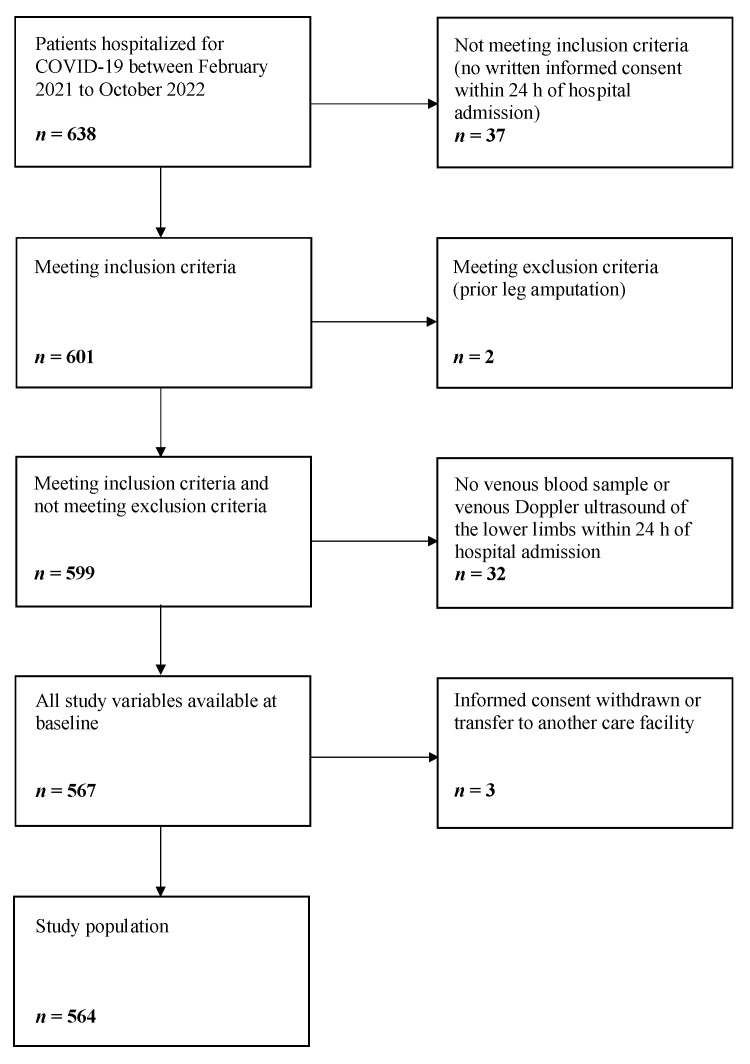
Selection of the study population.

A total of 564 COVID-19 patients (290 (51%) men, mean age of 74 ± 17 years) were consecutively enrolled (Figure 1).

At hospital admission, 130 (23%), 130 (23%), and 304 (54%) patients had mild, moderate, and severe COVID-19, respectively. Three hundred and twenty-three (57%) patients had received at least one dose of an anti-SARS-CoV-2 vaccine, while 14 (2%) had had a previous diagnosis of SARS-CoV-2 infection. Three hundred and fifty-two patients (62%) had a PP score value of ≥4, indicative of a high risk of VTE. The median Lp(a) level was 13 (10–27) mg/dL. Lp(a) tertiles were as follows: Lp(a) ≤ 10 mg/dL (first tertile), Lp(a) 11–21 mg/dL (second tertile), and Lp(a) > 21 mg/dL (third tertile). One hundred and twenty-three (21%) and 51 (9%) patients had Lp(a) > 30 mg/dL and Lp(a) > 50 mg/dL, respectively. The median D-dimer level was 1040 (604–2040) ng/mL. The median CRP, procalcitonin, and WBC levels were 4.4 (1.4–9.5) mg/dL, 0.13 (0.08–0.29) ng/mL, and 7.25 (5.1–10.4) × 1000/μL, respectively. Characteristics of the study population, categorized according to the median Lp(a) levels (≤versus > 13 mg/dL), are shown in Table 1. Characteristics of the study population, categorized according to Lp(a) ≤ versus > 30 mg/dL, Lp(a) ≤ versus > 50 mg/dL, and Lp(a) tertiles, are shown in Tables S1–S3.

### 3.2. Clinical Course

Corticosteroid treatment (6 mg of dexamethasone daily) was administered to 377 (67%) patients. Antiviral therapy with remdesivir was prescribed to 181 (32%) patients fulfilling the prescription criteria of the Italian drug agency (AIFA). Overall, anticoagulant therapy was administered to 473 (84%) patients (359 (64%) patients started thromboembolism prophylaxis with low-molecular-weight heparin (LMWH) at hospital admission, while 114 (20%) patients started/continued full anticoagulant therapy with either LMWH, vitamin K antagonists (VKAs), or direct oral anticoagulants (DOACs) during the hospital stay, depending on the underlying medical conditions requiring anticoagulation and concomitant diseases). Respiratory support was provided via NIV.

During the hospital stay, 72 thrombotic events (*n* = 28 DVT, *n* = 5 SVT, *n* = 12 EP, *n* = 14 stroke/TIA, *n* = 10 ACS, and *n* = 3 CLI) were diagnosed in 64 (11%) patients (27 (5%) patients were diagnosed with at least one arterial thrombotic event and 39 (7%) patients with at least one venous thrombotic event). The characteristics of patients who were diagnosed with thrombotic events, either arterial and venous combined or separated, are shown in Tables S4–S6. The rates of thrombotic events, either arterial and venous combined or separated, were significantly lower among patients who started thromboembolism prophylaxis with LMWH at hospital admission as compared to those who did not (thrombotic events: 5% versus 22%, *p* < 0.001; arterial thrombotic events: 3% versus 8%, *p* = 0.003; and venous thrombotic events: 3% versus 14%, *p* < 0.001). Instead, they did not differ significantly according to in-hospital therapy with corticosteroids or remdesivir (*p* > 0.05 for all comparisons).

During the hospital stay, 15 (3%) patients were admitted to the ICU, 70 (12%) patients died, and 83 (15%) patients met the composite end point of ICU admission/in-hospital death. The median time from hospital admission to ICU admission was 5 (2–8) days, while the median time from hospital admission to death was 11 (6–17) days. Characteristics of patients who were admitted to the ICU/died are shown in Table S7. Rates of ICU admission/in-hospital death did not differ significantly according to thromboembolism prophylaxis with in-hospital anticoagulant therapy. Instead, they were significantly higher among patients who were treated with corticosteroids (19 versus 5%, *p* < 0.001) as compared to those who were not, and significantly lower among patients treated with remdesivir (8% versus 18%, *p* = 0.003) as compared to those who were not.

### 3.3. Lp(a) and Biomarkers of Thrombo-Inflammation

No significant correlation was found between Lp(a) and either D-dimer (rho = 0.094, *p* = 0.052), CRP (rho = 0.022, *p* = 0.609), procalcitonin (rho = −0.062, *p* = 0.160), or WBC (rho = −0.011, *p* = 0.802) levels. D-dimer levels did not differ significantly according to Lp(a) ≤ versus > the median value (*p* = 0.088), Lp(a) ≤ versus > 30 mg/dL (*p* = 0.103), Lp(a) ≤ versus > 50 mg/dL (*p* = 0.083), or Lp(a) tertiles (*p* for trend = 0.089) (Figure S1a–d). No significant difference was observed in CRP, procalcitonin, and WBC levels according to Lp(a) ≤ versus > the median value (*p* = 0.974, *p* = 0.283, and *p* = 0.828 for CRP, procalcitonin, and WBC, respectively), Lp(a) ≤ versus > 30 mg/dL (*p* = 0.460, *p* = 0.687, and *p* = 0.696 for CRP, procalcitonin, and WBC, respectively), Lp(a) ≤ versus > 50 mg/dL (*p* = 0.500, *p* = 0.842, and *p* = 0.742 for CRP, procalcitonin, and WBC, respectively) or Lp(a) tertiles (*p* for trend = 0.856, *p* for trend = 0.264, and *p* for trend = 0.988 for CRP, procalcitonin, and WBC, respectively) (Figure S1e–p).

### 3.4. Lp(a) and Thrombotic Events

**Figure 2:**
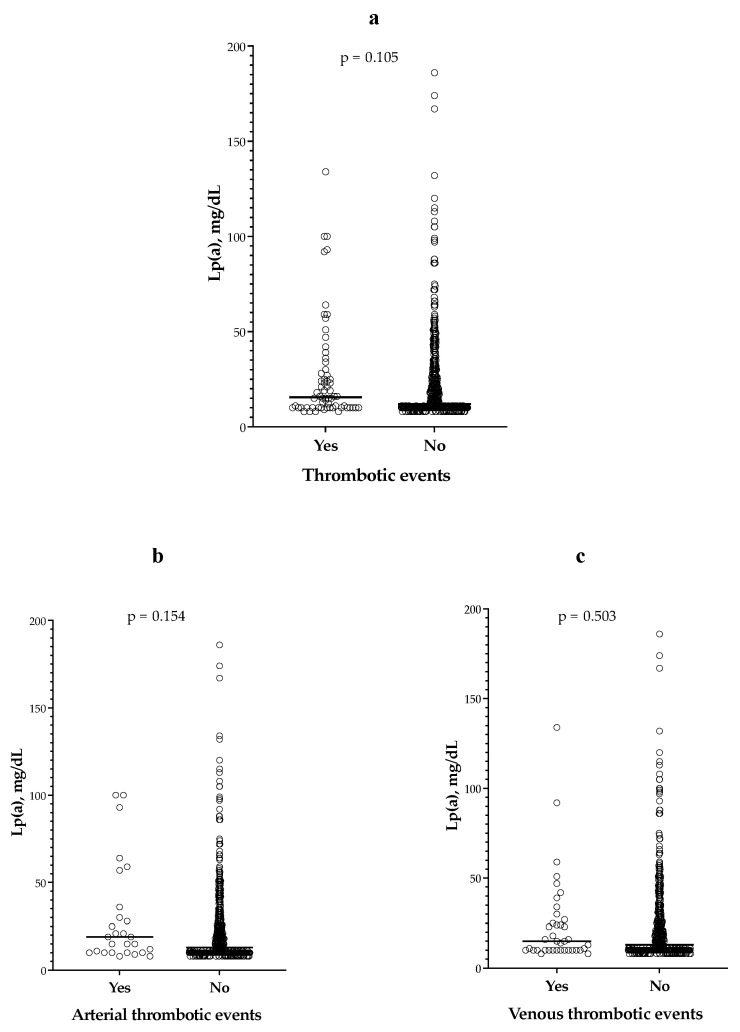
Lp(a) levels according to the occurrence of thrombotic events. (**a**) Lp(a) levels according to the occurrence of thrombotic events (arterial and venous thrombotic events combined). (**b**) Lp(a) levels according to the occurrence of arterial thrombotic events. (**c**) Lp(a) levels according to the occurrence of venous thrombotic events.

Lp(a) levels were comparable between patients who were diagnosed with thrombotic events, either arterial and venous combined or separated, and those who were not (Figure 2).

**Figure 3:**
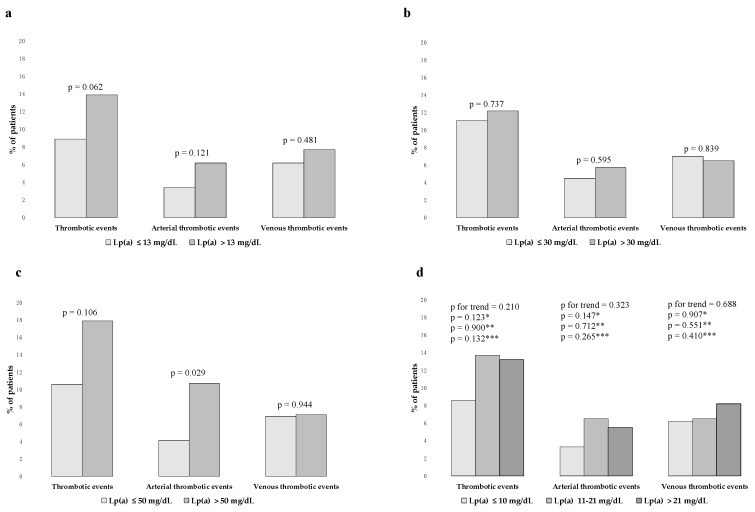
Rates of thrombotic events according to Lp(a) levels. (**a**) Rates of thrombotic events (either arterial and venous combined or separated) according to Lp(a) ≤ versus > the median value. (**b**) Rates of thrombotic events (either arterial and venous combined or separated) according to Lp(a) ≤ versus > 30 mg/dL. (**c**) Rates of thrombotic events (either arterial and venous combined or separated) according to Lp(a) ≤ versus > 50 mg/dL. (**d**) Rates of thrombotic events (either arterial and venous combined or separated) according to Lp(a) tertiles. * First versus second tertile; ** second versus third tertile; and *** first versus third tertile.

The rates of thrombotic events, either arterial and venous combined or separated, did not differ according to Lp(a) ≤ versus > the median value (Figure 3a), nor according to Lp(a) ≤ versus > 30 mg/dL (Figure 3b). A higher rate of arterial thrombotic events was observed among patients with Lp(a) > 50 mg/dL as compared to those with Lp(a) ≤ 50 mg/dL (Figure 3c). Comparable rates of thrombotic events, either arterial and venous combined or separated, emerged across Lp(a) tertiles (Figure 3d). The ORs for the occurrence of thrombotic events, either arterial and venous combined or separated, according to Lp(a) levels, are shown in Table 2.

### 3.5. Lp(a) and the Composite End Point of ICU Admission/in-Hospital Death

**Figure 4:**
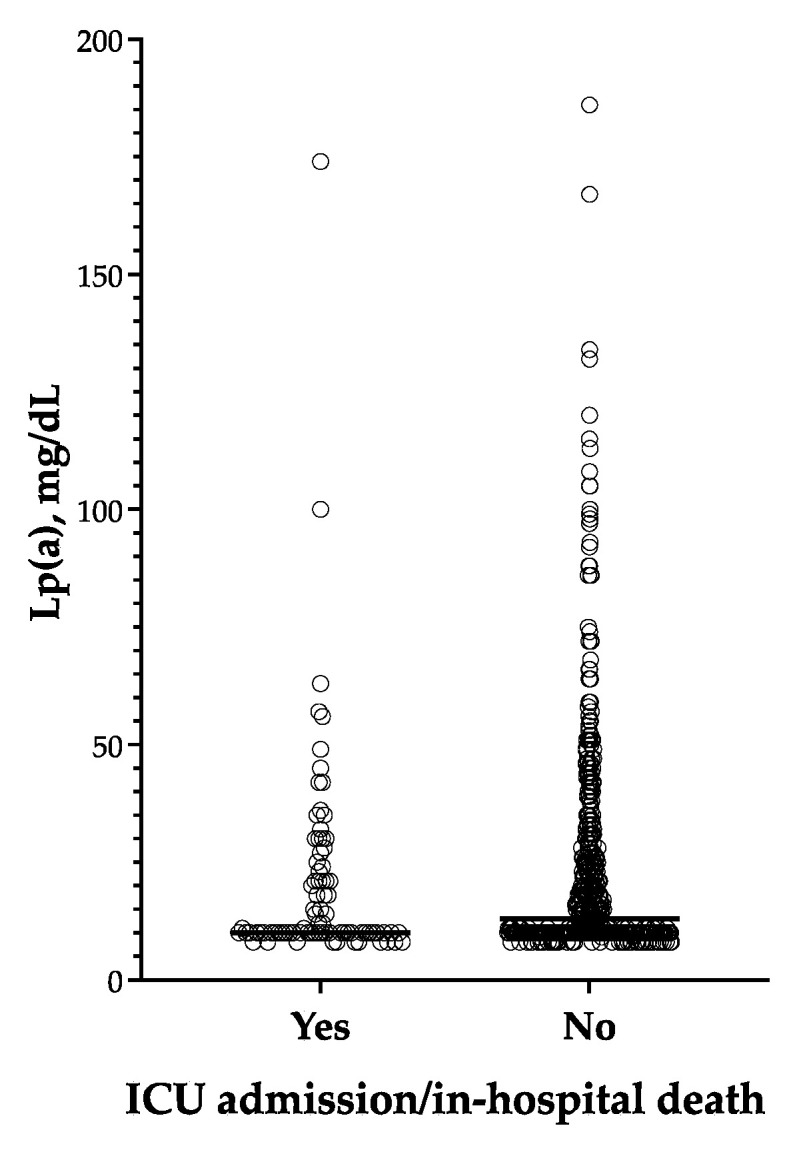
Lp(a) levels according to the composite end point of ICU admission/in-hospital death.

There was no significant difference in Lp(a) levels between patients who were admitted to the ICU/died and those who were not admitted to the ICU/were discharged alive (Figure 4).

**Figure 5:**
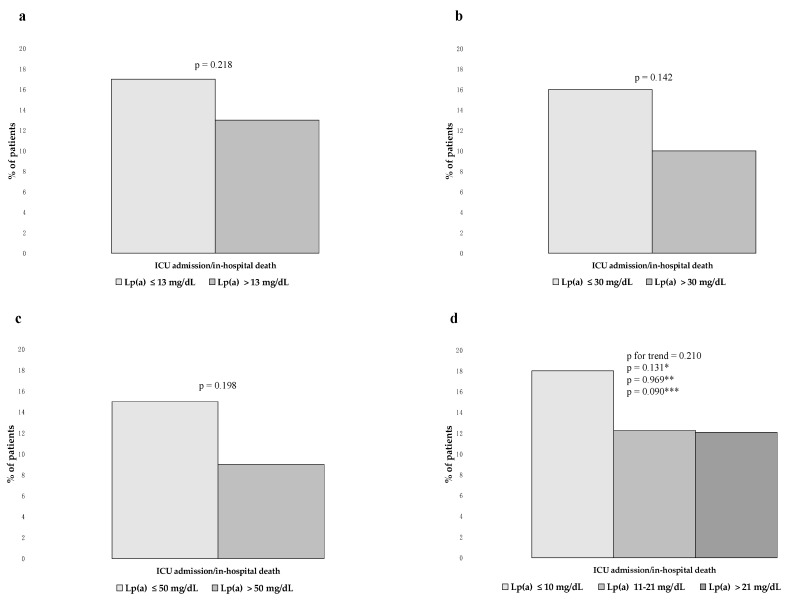
Rates of ICU admission/in-hospital death according to Lp(a) levels. (**a**) Rates of ICU admission/in-hospital death according to Lp(a) ≤ versus > the median value. (**b**) Rates of ICU admission/in-hospital death according to Lp(a) ≤ versus > 30 mg/dL. (**c**) Rates of ICU admission/in-hospital death according to Lp(a) ≤ versus > 50 mg/dL. (**d**) Rates of ICU admission/in-hospital death according to Lp(a) tertiles. * first versus second tertile; ** second versus third tertile; and *** first versus third tertile.

Rates of ICU admission/in-hospital death did not differ significantly according to Lp(a) ≤ versus > the median value, Lp(a) ≤ versus > 30 mg/dL, and Lp(a) ≤ versus > 50 mg/dL (Figure 5a–c). Additionally, they did not differ significantly across Lp(a) tertiles (Figure 5d). The HRs for ICU admission/in-hospital death according to Lp(a) levels are shown in Table 3.

### 3.6. Exploratory Analyses in the Subgroup of Patients with Severe COVID-19

In the subgroup of patients with severe COVID-19, Lp(a) levels did not differ significantly between patients who were diagnosed with thrombotic events, either arterial and venous combined or separated, and those who were not (*p* = 0.218 for arterial and venous thrombotic events combined, *p* = 0.143 for arterial thrombotic events, and *p* = 0.647 for venous thrombotic events), nor between patients who were admitted to the ICU/died and those who were not admitted to the ICU/were discharged alive (*p* = 0.933). In addition, neither rates of thrombotic events, either arterial and venous combined or separated, nor rates of ICU admission/in-hospital death differed significantly according to Lp(a) levels (Figure S2 and Figure S3, respectively). The ORs for the occurrence of thrombotic events, either arterial and venous combined or separated, according to Lp(a) levels in the subgroup of patients with severe COVID-19, are shown in Table S8. The HRs for ICU admission/in-hospital death according to Lp(a) levels in the subgroup of patients with severe COVID-19 are shown in Table S9.

## 4. Discussion

In our study on 564 patients hospitalized for COVID-19, we did not observe any significant association between Lp(a) levels and the occurrence of thrombotic events, either arterial or venous, and the composite end point of ICU admission/in-hospital death. In addition, circulating Lp(a) levels at hospital admission were not significantly associated with biomarkers of thrombo-inflammation.

The lack of an association between Lp(a) and venous thrombotic events observed in our study fits into the context of inconsistent literature data. Pawlos et al. reported comparable rates of pulmonary embolism between COVID-19 patients with Lp(a) < 30 mg/dL and those with Lp(a) ≥ 30 mg/dL [26]. In contrast, Nurmohamed et al. showed a three-fold higher risk of venous thromboembolism with increasing Lp(a) levels during the hospital stay [28]. Of note, the recorded rates of venous thrombotic events were somewhat lower in our study (7%) as compared to the study by Nurmohamed et al. (30%) [28], despite anticoagulant therapy being part of in-hospital therapeutic strategies against COVID-19 in both studies. Thus, it cannot be excluded that other clinical conditions that reduce the risk of venous thrombosis, beyond in-hospital anticoagulation, may have masked a possible association between Lp(a) and the occurrence of venous thrombotic events in our study. Nonetheless, we cannot support a predictive role for Lp(a) in the pathway of venous thrombotic events in the context of COVID-19. In line with this point, further uncertainty emerges regarding the association between Lp(a) and incident venous thrombotic events also in the general population [40,41,42].

It is noteworthy that we observed a significantly higher risk of arterial thrombotic events in patients with Lp(a) levels > 50 mg/dL as compared to those with Lp(a) ≥ 50 mg/dL; however, the small sample of patients with Lp(a) levels > 50 mg/dL (10% of the study population) may limit the reliability of this result. In addition, the association between Lp(a) levels > 50 mg/dL and the occurrence of arterial thrombotic events was not significant after adjusting for potential confounders. Thus, it remains unclear whether extremely high Lp(a) levels may be associated with an increased short-term risk of arterial thrombotic events among patients hospitalized for COVID-19.

Our finding of a non-significant association between Lp(a) and in-hospital clinical outcomes agrees with the results from most of the previous studies [28,29,30,31]. In addition, it is consistent with our report of a non-significant association between Lp(a) levels and either biomarkers of thrombo-inflammation or the occurrence of thrombotic events, which are recognized as major determinants of COVID-19 in-hospital prognoses [43,44,45].

Our observation of a non-significant association between D-dimer and Lp(a) levels is in contrast with the results of two previous studies [26,29]. Indeed, Kaltoft et al. previously reported that two-fold higher D-dimer levels were associated with 14% higher Lp(a) levels among 211 patients admitted with COVID-19 [29]. Additionally, Pawlos et al. found significantly higher D-dimer levels in the presence of Lp(a) ≥ 30 mg/dL as compared to Lp(a) < 30 mg/dL among 124 patients hospitalized for COVID-19 [26]. Of note, in our cohort 94 (17%) patients were taking oral anticoagulant therapy prior to hospitalization, which may have confounded, at least in part, a possible association between Lp(a) and D-dimer as a marker of plasma thrombotic activity; however, Lp(a) was not a predictor of D-dimer levels, even after adjusting for the potential confounding effect of preadmission oral anticoagulant therapy. Thus, whether Lp(a) may drive increased plasma thrombotic activity in COVID-19 remains arguable [22,23,46,47].

The non-significant association that we found between Lp(a) and circulating inflammatory biomarkers was not surprising based on variable results from previous studies [26,27,29]. In line with our study, Pawlos et al. reported that circulating levels of CRP and procalcitonin did not differ significantly between Lp(a) < 30 mg/dL and Lp(a) ≥ 30 mg/dL among 124 patients hospitalized for COVID-19 [26]. Additionally, Lippi et al. did not describe a significant association between Lp(a) and CRP among 50 patients hospitalized for COVID-19 [27]. In contrast, Kaltoft et al. reported 5.2% and 8.7% lower Lp(a) levels in the presence of two-fold higher CRP and procalcitonin levels in 211 patients admitted with COVID-19 [29]. An explanatory hypothesis for such discrepancies could be that the relationship between Lp(a) and inflammatory biomarkers may be influenced by different clinical variables and drugs prescribed prior to hospitalization, thereby being somehow unpredictable across different populations of patients hospitalized for COVID-19. In this regard, attention should be paid to possible anti-SARS-CoV-2 vaccine-mediated attenuation of immune–inflammatory responses in breakthrough cases. Accordingly, the only previous study showing a significant association between Lp(a) and inflammatory biomarkers was performed in a cohort of unvaccinated COVID-19 patients with slightly higher inflammatory biomarkers as compared to other studies and our study [27]. Nonetheless, in our cohort Lp(a) was not a predictor of either CRP, procalcitonin, or WBC levels even after adjusting for the potential confounding effect of a previous anti-SARS-CoV-2 vaccine. Thus, a possible pathophysiological link between Lp(a) and systemic inflammation in COVID-19 remains questionable [22,23,48].

Overall, the results of our study question the hypothesis that Lp(a) plays an important pathogenic role in the evolution of COVID-19 towards its most severe forms. From a clinical perspective, they imply that Lp(a) cannot be used as a prognostic biomarker in COVID-19. Moreover, they suggest that Lp(a) is unlikely as a possible therapeutic target in COVID-19. It is noteworthy that there is evidence from randomized clinical trials of better in-hospital outcomes in COVID-19 patients treated with either tocilizumab or anti-PCSK9 monoclonal antibodies [49,50,51], which are recognized pharmacological strategies with the ability to reduce Lp(a) levels by ~30% beyond their main therapeutic effects [52,53]; however, no data are available on the relationship between Lp(a) reduction by either tocilizumab or anti-PCSK9 monoclonal antibodies and the clinical outcomes of patients hospitalized for COVID-19 [49,50,51]. Thus, the available intervention studies also do not substantiate any rationale for targeting Lp(a) to improve COVID-19 prognoses.

Some strengths of our study should be acknowledged. First, this is the largest observational study to explore the possible predictive role of Lp(a) in thrombotic events and clinical outcomes among patients hospitalized for COVID-19. Second, it is the first study to have analyzed the association between Lp(a) levels and the occurrence of both venous and arterial thrombotic events among COVID-19 patients. Third, at the time of the data analysis, follow-up data were available for all patients and no clinical end point was censored.

Some limitations of our study should also be considered. First, the use of an isoform-sensitive assay for Lp(a) measurement might have confounded, at least in part, the observed results by underestimating or overestimating circulating Lp(a) concentrations in the presence of small or large apo(a) isoforms, respectively [54]. Second, Lp(a) and biomarkers of thrombo-inflammation were only determined at the study baseline; thus, the association between their temporal changes during the hospital stay and clinical end points was not evaluated. Third, the absence of a long-term follow-up for patients who were discharged alive only allowed us to assess Lp(a) as a predictor of in-hospital thrombotic events and prognosis.

In conclusion, our study shows that Lp(a) cannot predict the clinical course of COVID-19 nor is associated with biomarkers of thrombo-inflammation. Thus, Lp(a) is unlikely to be a possible prognostic biomarker or therapeutic target in the context of COVID-19.

## Figures and Tables

**Table 1:** Characteristics of the study population according to Lp(a) levels ≤ versus > the median value (i.e., 13 mg/dL).

	Lp(a) ≤ 13 mg/dL	Lp(a) > 13 mg/dL	*p*
Age, years	73 ± 17	76 ± 16	0.069
Male sex, %	51	51	0.881
BMI, Kg/m^2^	26 ± 5	26 ± 4	0.102
Current smoking, %	7	6	0.623
Hypertension, %	62	71	0.033
Type 2 diabetes, %	21	24	0.360
CKD, %	16	19	0.366
ASCVD, %	19	28	0.012
AF, %	19	19	0.959
Previous VTE, %	6	7	0.711
ACE inhibitors, %	28	25	0.497
ARBs, %	12	15	0.198
BBs, %	29	37	0.049
CCBs, %	22	25	0.506
Diuretics, %	36	44	0.068
Oral anticoagulants, %	16	17	0.572
Antiplatelets, %	22	32	0.006
Oral hypoglycemic drugs, %	12	11	0.804
Insulin, %	11	13	0.503
Statins, %	19	30	0.002
Anti-SARS-CoV-2 vaccine, %	60	65	0.287
PaO_2_/FiO_2_ < 300, %	53	48	0.310
Total cholesterol, mg/dL	149 ± 40	157 ± 42	0.043
LDL cholesterol, mg/dL	87 ± 32	94 ± 34	0.016
HDL cholesterol, mg/dL	40 ± 17	40 ± 14	0.729
Triglycerides, mg/dL	105 (73–144)	101 (79–130)	0.676
CCI	5 (3–7)	5 (4–7)	0.053
SOFA score	2 (2–4)	2 (1–4)	0.636
PP score	5 (3–6)	5 (3–7)	0.257

Acronyms. AF, atrial fibrillation; ACE, angiotensin-converting enzyme; ASCVD, atherosclerotic cardiovascular disease; ARBs, angiotensin receptor blockers; BBs, beta blockers; BMI, body mass index; CCBs, calcium channel blockers; CCI, Charlson Comorbidity Index; CKD, chronic kidney disease; FiO_2_, fraction of inspired oxygen; HDL, high-density lipoproteins; LDL, low-density lipoproteins; Lp(a), lipoprotein(a); PaO_2_, partial pressure of oxygen; PP, Padua prediction; SOFA, sequential organ failure assessment; and VTE, venous thromboembolism. The values of variables are expressed as mean ± SD, median (interquartile range), or percentages; the *p*-values are for two-group comparisons.

**Table 2:** Odds ratios for the occurrence of thrombotic events according to Lp(a) levels.

		Model 1	Model 2	Model 3	Model 4
Dependent variable: thrombotic events	Lp(a), mg/dL	1.006 (95%CI 0.997–1.016)	1.007 (95%CI 0.997–1.017)	* 1.010 (95%CI 0.999–1.021)	° 1.009 (95%CI 0.998–1.020)
	Lp(a) > 13 mg/dL	1.648 (95%CI 0.971–2.797)	1.568 (95%CI 0.914–2.689)	* 1.641 (95%CI 0.925–2.910)	° 1.561 (95%CI 0.862–2.827)
	Lp(a) > 30 mg/dL	1.111 (95%CI 0.600–2.058)	1.066 (95%CI 0.569–1.997)	* 1.192 (95%CI 0.613–2.317)	° 1.153 (95%CI 0.586–2.267)
	Lp(a) > 50 mg/dL	1.828 (95%CI 0.872–3.830)	1.841 (95%CI 0.862–3.935)	* 2.092 (95%CI 0.921–4.750)	° 2.015 (95%CI 0.879–4.623)
	Lp(a) tertiles	1.264 (95%CI 0.935–1.707)	1.040 (95%CI 1.018–1.063)	* 1.305 (95%CI 0.935–1.822)	° 1.251 (95%CI 0.889–1.760)
Dependent variable: arterial thrombotic events	Lp(a), mg/dL	1.009 (95%CI 0.997–1.022)	1.011 (95%CI 0.998–1.024)	** 1.012 (95%CI 0.998–1.026)	°° 1.011 (95%CI 0.997–1.026)
	Lp(a) > 13 mg/dL	1.866 (95%CI 0.839–4.150)	1.767 (95%CI 0.785–3.979)	** 1.702 (95%CI 0.740–3.912)	°° 1.528 (95%CI 0.631–3.697)
	Lp(a) > 30 mg/dL	1.270 (95%CI 0.524–3.078)	1.219 (95%CI 0.497–2.994)	** 1.240 (95%CI 0.492–3.125)	°° 1.276 (95%CI 0.494–3.299)
	Lp(a) > 50 mg/dL	2.783 (95%CI 1.073–7.216)	2.853 (95%CI 1.072–7.593)	** 2.974 (95%CI 1.082–8.173)	°° 3.228 (95%CI 1.148–9.079)
	Lp(a) tertiles	1.291 (95%CI 0.825–2.020)	1.265 (95%CI 0.799–2.002)	** 1.245 (95%CI 0.772–2.006)	°° 1.138 (95%CI 0.688–1.881)
Dependent variable: venous thrombotic events	Lp(a), mg/dL	1.002 (95%CI 0.989–1.015)	1.002 (95%CI 0.989–1.015)	*** 0.997 (95%CI 0.978–1.017)	°°° 0.672 (95%CI 0.976–1.016)
	Lp(a) > 13 mg/dL	1.264 (95%CI 0.658–2.427)	1.202 (95%CI 0.623–2.322)	*** 1.068 (95%CI 0.477–2.392)	°°° 1.082 (95%CI 0.474–2.474)
	Lp(a) > 30 mg/dL	0.920 95%CI 0.412–2.056)	0.888 (95%CI 0.395–1.995)	*** 0.971 (95%CI 0.372–2.537)	°°° 0.905 (95%CI 0.339–2.418)
	Lp(a) > 50 mg/dL	1.040 (95%CI 0.355–3.041)	1.023 (95%CI 0.347–3.015)	*** 0.798 (95%CI 0.205–3.110)	°°° 0.699 (95%CI 0.174–2.809)
	Lp(a) tertiles	1.291 (95%CI 0.825–2.020)	1.145 (95%CI 0.783–1.676)	*** 1.055 (95%CI 0.661–1.685)	°°° 1.056 (95%CI 0.656–1.699)

Model 1: unadjusted. Model 2: adjusted for age and sex. Model 3: * adjusted for age, sex, ASCVD, previous VTE, and in-hospital thromboembolism prophylaxis with LMWH; ** adjusted for age, sex, ASCVD, and in-hospital thromboembolism prophylaxis with LMWH; *** adjusted for age, sex, previous VTE, preadmission oral anticoagulants, PP score, D-dimer, and in-hospital thromboembolism prophylaxis with LMWH; ° adjusted for age, sex, ASCVD, previous VTE, anti-SARS-CoV-2 vaccine, and in-hospital thromboembolism prophylaxis with LMWH; °° adjusted for age, sex, ASCVD, anti-SARS-CoV-2 vaccine, and in-hospital thromboembolism prophylaxis with LMWH; °°° adjusted for age, sex, previous VTE, preadmission oral anticoagulants, anti-SARS-CoV-2 vaccine, PP score, D-dimer, and in-hospital thromboembolism prophylaxis with LMWH.

**Table 3:** Hazard ratios for ICU admission/in-hospital death according to Lp(a) levels.

		Model 1	Model 2	Model 3	Model 4
Dependent variable: ICU admission/in-hospital death	Lp(a), mg/dL	0.997 (95%CI 0.986–1.009)	0.997 (95%CI 0.986–1.008)	1.006 (95%CI 0.992–1.019)	1.004 (95%CI 0.990–1.019)
	Lp(a) > 13 mg/dL	0.817 (95%CI 0.526–1.269)	0.798 (95%CI 0.514–1.239)	1.212 (95%CI 0.602–2.439)	1.123 (95%CI 0.548–2.298)
	Lp(a) > 30 mg/dL	0.773 (95%CI 0.426–1.403)	0.746 (95%CI 0.410–1.355)	0.744 (95%CI 0.311–1.781)	0.654 (95%CI 0.261–1.639)
	Lp(a) > 50 mg/dL	0.748 (95%CI 0.301–1.858)	0.735 (95%CI 0.296–1.827)	1.060 (95%CI 0.310–3.624)	1.019 (95%CI 0.292–3.559)
	Lp(a) tertiles	0.865 (95%CI 0.666–1.122)	0.855 (95%CI 0.657–1.114)	1.037 (95%CI 0.699–1.538)	1.021 (95%CI 0.684–1.525)

Model 1: unadjusted. Model 2: adjusted for age and sex. Model 3: adjusted for age, sex, BMI, CKD, AF, preadmission ACE inhibitors, preadmission diuretics, preadmission insulin, SOFA score, PP score, CRP, D-dimer, LDL cholesterol, HDL cholesterol, triglycerides, in-hospital corticosteroids, and in-hospital remdesivir. Model 4: adjusted for age, sex, BMI, CKD, AF, anti-SARS-CoV-2 vaccine, preadmission ACE inhibitors, preadmission diuretics, preadmission insulin, SOFA score, PP score, CRP, D-dimer, LDL cholesterol, HDL cholesterol, triglycerides, in-hospital corticosteroids, and in-hospital remdesivir.

## Data Availability

The data presented in this study are available on request from the corresponding author.

## Supplementary Materials

The following supporting information can be downloaded at: https://www.mdpi.com/article/10.3390/jcm12103543/s1, Table S1: Baseline characteristics of the study population according to Lp(a) ≤ versus > 30 mg/dL; Table S2: Baseline characteristics of the study population according to Lp(a) ≤ versus > 50 mg/dL; Table S3: Baseline characteristics of the study population according to Lp(a) tertiles; Table S4: Baseline characteristics of the study population according to the occurrence of thrombotic events; Table S5: Baseline characteristics of the study population according to the occurrence of arterial thrombotic events; Table S6: Baseline characteristics of the study population according to the occurrence of venous thrombotic events; Table S7: Baseline characteristics of the study population according to ICU admission/in-hospital death; Table S8: Odds ratios for the occurrence of thrombotic events according to Lp(a) levels in the subgroup of patients with severe COVID-19; Table S9: Hazard ratios for ICU admission/in-hospital death according to Lp(a) levels in the subgroup of patients with severe COVID-19; Figure S1: Biomarkers of thrombo-inflammation according to Lp(a) levels; Figure S2: Rates of thrombotic events according to Lp(a) levels in the subgroup of patients with severe COVID-19; Figure S3: Rates of ICU admission/in-hospital death according to Lp(a) levels in the subgroup of patients with severe COVID-19.
