# Supplementary material for: Lipoprotein(a) Does Not Predict Thrombotic Events and In-Hospital Outcomes in Patients with COVID-19
Source: J Clin Med. 2023 May 18;12(10):3543. doi: 10.3390/jcm12103543 (PMC10218794; doi:10.3390/jcm12103543)
Supplement: Supplementary file 1 [file jcm-12-03543-s001.zip › jcm-2390462-supplementary.pdf]

Supplementary Materials

	Lp(a) ≤ 30 mg/dL	Lp(a) > 30 mg/dL	p
**Age, years**	74 ± 17	75 ± 17	0.730
**Male sex, %**	51	52	0.878
**BMI, Kg/m^2**	26 ± 5	26 ± 4	0.322
**Current smoking, %**	6	6	0.723
**Hypertension, %**	67	63	0.442
**Type 2 diabetes, %**	23	22	0.783
**CKD, %**	16	24	0.034
**ASCVD, %**	21	32	0.016
**AF, %**	19	20	0.751
**Previous VTE, %**	6	6	0.977
**ACE inhibitors, %**	28	23	0.340
**ARBs, %**	13	15	0.670
**BBs, %**	33	36	0.516
**CCBs, %**	23	26	0.154
**Diuretics, %**	40	40	0.952
**Oral anticoagulants, %**	17	16	0.891
**Antiplatelets, %**	25	34	0.042
**Oral hypoglycemic drugs, %**	13	7	0.087
**Insulin, %**	12	10	0.611
**Statins, %**	23	29	0.145
**Anti-SARS-CoV-2 vaccine, %**	61	66	0.310

**PaO_2/FiO_2 < 300, %**	52	43	0.093
**Total cholesterol, mg/dL**	151 ± 41	159 ±42	0.089
**LDL cholesterol, mg/dL**	89 ± 34	95 ±31	0.115
**HDL cholesterol, mg/dL**	40 ± 16	39 ± 14	0.691
**Triglycerides, mg/dL**	103 (74-139)	101 (80-132)	0.869
**CCI**	5 (3-7)	5 (4-8)	0.142
**SOFA score**	2 (2-4)	3 (1-4)	0.980
**PP score**	5 (3-6)	5 (3-7)	0.082

Table S1. Baseline characteristics of the study population according to Lp(a) ≤ versus > 30 mg/dL.

AF, atrial fibrillation; ACE, angiotensin converting enzyme; ASCVD, atherosclerotic cardiovascular disease; ARBs, angiotensin receptor blockers; BBs, beta blockers; BMI, body mass index; CCBs, calcium channel blockers; CCI, Charlson Comorbidity Index; CKD, chronic kidney disease; FiO2, fraction of inspired oxygen; HDL, high-density lipoprotein; LDL, low-density lipoprotein; Lp(a), lipoprotein(a); PaO2, partial pressure of oxygen; PP, Padua prediction; SOFA, Sequential Organ Failure Assessment; VTE, venous thromboembolism. Values of variables are expressed as mean ± SD, median (interquartile range), or percentages.

	Lp(a) ≤ 50 mg/dL	Lp(a) > 50 mg/dL	p
**Age, years**	74 ± 17	75 ± 16	0.662
**Male sex, %**	51	50	0.823
**BMI, Kg/m^2**	26 ± 5	26 ± 5	0.580
**Current smoking, %**	6	7	0.806
**Hypertension, %**	67	61	0.350
**Type 2 diabetes, %**	22	25	0.690
**CKD, %**	17	27	0.068
**ASCVD, %**	22	36	0.024
**AF, %**	19	18	0.769
**Previous VTE, %**	6	11	0.186
**ACE inhibitors, %**	27	25	0.729
**ARBs, %**	13	18	0.312
**BBs, %**	33	36	0.690
**CCBs, %**	23	27	0.763
**Diuretics, %**	40	43	0.654
**Oral anticoagulants, %**	17	16	0.900
**Antiplatelets, %**	25	43	0.005
**Oral hypoglycemic drugs, %**	12	7	0.263
**Insulin, %**	12	12	0.880
**Statins, %**	23	39	0.006
**Anti-SARS-CoV-2 vaccine, %**	62	60	0.754
**PaO_2/FiO_2 < 300, %**	51	42	0.246

**Total cholesterol, mg/dL**	151 ± 41	163 ± 42	0.062
**LDL cholesterol, mg/dL**	98 ± 32	90 ± 33	0.107
**HDL cholesterol, mg/dL**	40 ± 15	41 ± 14	0.752
**Triglycerides, mg/dL**	102 (75-138)	107 (84-132)	0.519
**CCI**	5 (3-7)	6 (4-8)	0.132
**SOFA score**	2 (2-4)	3 (1-3)	0.880
**PP score**	5 (3-6)	6 (3-7)	0.040

Table S2. Baseline characteristics of the study population according to according to Lp(a) ≤ versus > 50 mg/dL.

AF, atrial fibrillation; ACE, angiotensin converting enzyme; ASCVD, atherosclerotic cardiovascular disease; ARBs, angiotensin receptor blockers; BBs, beta blockers; BMI, body mass index; CCBs, calcium channel blockers; CCI, Charlson Comorbidity Index; CKD, chronic kidney disease; FiO2, fraction of inspired oxygen; HDL, high-density lipoprotein; LDL, low-density lipoprotein; Lp(a), lipoprotein(a); PaO2, partial pressure of oxygen; PP, Padua; SOFA, Sequential Organ Failure Assessment; VTE, venous thromboembolism.

Values of variables are expressed as mean ± SD, median (interquartile range), or percentages.

	Lp(a) ≤ 10 mg/dL (1st quartile)	Lp(a) 11-20 mg/dL (2nd quartile)	Lp(a) > 20 mg/dL (3rd quartile)	p for trend
**Age, years**	73 ± 17	76 ± 16	75 ± 17	0.119
**Male sex, %**	51	52	50	0.941
**BMI, Kg/m^2**	26 ± 5	26 ± 4	26 ± 4	0.218
**Current smoking, %**	7	6	6	0.870
**Hypertension, %**	62	72	67	0.169
**Type 2 diabetes, %**	20	26	24	0.471
**CKD, %**	16	16	21	0.318
**ASCVD, %**	19	23	30	0.045
**AF, %**	20	21	16	0.497
**Previous VTE, %**	6	5	8	0.490
**ACE inhibitors, %**	29	24	26	0.498
**ARBs, %**	11	16	15	0.221
**BBs, %**	29	43	32	0.015
**CCBs, %**	21	27	24	0.485
**Diuretics, %**	37	42	42	0.540
**Oral anticoagulants, %**	16	21	13	0.134
**Antiplatelets, %**	22	28	33	0.035
**Oral hypoglycemic drugs, %**	11	14	10	0.667
**Insulin, %**	11	11	13	0.798
**Statins, %**	17	28	31	0.001
**Anti-SARS-CoV-2 vaccine, %**	59	67	63	0.310

**PaO_2/FiO_2 < 300, %**	53	50	47	0.475
**Total cholesterol, mg/dL**	148 ± 41	153 ± 38	159 ± 45	0.756
**LDL cholesterol, mg/dL**	86 ± 33	91 ± 32	96 ± 34	0.607
**HDL cholesterol, mg/dL**	40 ± 18	39 ± 12	40 ±14	0.934
**Triglycerides, mg/dL**	104 (74-139)	100 (74-127)	103 (80-143)	0.558
**CCI**	5 (3-7)	5 (4-7)	5 (3-8)	0.334
**SOFA score**	2 (2-4)	2 (1-4)	2 (1-4)	0.902
**PP score**	5 (3-6)	4 (3-6)	5 (3-7)	0.360

Table S3. Baseline characteristics of the study population according to Lp(a) tertiles.
AF, atrial fibrillation; ACE, angiotensin converting enzyme; ASCVD, atherosclerotic cardiovascular disease; ARBs, angiotensin receptor blockers; BBs, beta blockers; BMI, body mass index; CCBs, calcium channel blockers; CCI, Charlson Comorbidity Index; CKD, chronic kidney disease; FiO2, fraction of inspired oxygen; HDL, high-density lipoprotein; LDL, low-density lipoprotein; Lp(a), lipoprotein(a); PaO2, partial pressure of oxygen; PP, Padua prediction; SOFA, Sequential Organ Failure Assessment; VTE, venous thromboembolism.

Values of variables are expressed as mean ± SD, median (interquartile range), or percentages.

	Thrombotic events	No thrombotic events	p
**Age, years**	82 ± 11	73 ± 17	< 0.001
**Male sex, %**	37	53	0.018
**BMI, Kg/m^2**	26 ± 4	26 ± 5	0.473
**Current smoking, %**	3	7	0.257
**Hypertension, %**	73	65	0.200
**Type 2 diabetes, %**	20	23	0.605
**CKD, %**	14	18	0.394
**ASCVD, %**	34	22	0.031
**AF, %**	20	19	0.832
**Previous VTE, %**	16	5	0.002
**ACE inhibitors, %**	28	27	0.822
**ARBs, %**	16	13	0.593
**BBs, %**	41	32	0.189
**CCBs, %**	26	23	0.753
**Diuretics, %**	47	39	0.238
**Oral anticoagulants, %**	12	17	0.342
**Antiplatelets, %**	41	25	0.009
**Oral hypoglycemic drugs, %**	9	12	0.538
**Insulin, %**	12	12	0.871
**Statins, %**	33	23	0.091
**Anti-SARS-CoV-2 vaccine, %**	71	61	0.404
**PaO_2/FiO_2 < 300, %**	55	50	0.423

**D-dimer, ng/mL**	1332 (712-3104)	996 (603-1909)	0.078
**CRP, mg/dL**	5 (1.8-9.8)	4.4 (1.4-9.4)	0.630
**Procalcitonin, ng/mL**	0.15 (0.10-0.32)	0.13 (0.08-0.29)	0.255
**WBC, X 1000/ µL**	7.7 (5.2-12)	7.1 (5.1-10.3)	0.668
**Total cholesterol, mg/dL**	153 ± 38	153 ± 42	0.997
**LDL cholesterol, mg/dL**	90 ± 32	91 ± 34	0.869
**HDL cholesterol, mg/dL**	39 ± 14	40 ± 15	0.517
**Triglycerides, mg/dL**	113 (78-155)	102 (76-133)	0.110
**CCI**	5 (3-8)	5 (3-7)	0.627
**SOFA score**	3 (2-4)	2 (1-4)	0.321
**PP score**	5 (3-7)	5 (3-6)	0.096

Table S4. Baseline characteristics of the study population according to the occurrence of thrombotic events.

AF, atrial fibrillation; ACE, angiotensin converting enzyme; ASCVD, atherosclerotic cardiovascular disease; ARBs, angiotensin receptor blockers; BBs, beta blockers; BMI, body mass index; CCBs, calcium channel blockers; CCI, Charlson Comorbidity Index; CKD, chronic kidney disease; CRP, C-reactive protein; FiO2, fraction of inspired oxygen; HDL, high-density lipoprotein; LDL, low-density lipoprotein; Lp(a), lipoprotein(a); PaO2, partial pressure of oxygen; PP, Padua prediction; SOFA, Sequential Organ Failure Assessment; VTE, venous thromboembolism; WBC, white blood cells.

Values of variables are expressed as mean ± SD, median (interquartile range), or percentages.

	Arterial thrombotic events	No arterial thrombotic events	p
**Age, years**	84 ± 7	74 ± 17	<0.001
**Male sex, %**	33	52	0.054
**BMI, Kg/m^2**	26 ± 3	26 ± 5	0.241
**Current smoking, %**	0	7	0.164
**Hypertension, %**	74	66	0.382
**Type 2 diabetes, %**	37	22	0.073
**CKD, %**	18	18	0.932
**ASCVD, %**	48	22	0.002
**AF, %**	33	18	0.059
**Previous VTE, %**	11	6	0.328
**ACE inhibitors, %**	26	27	0.902
**ARBs, %**	22	13	0.173
**BBs, %**	48	32	0.094
**CCBs, %**	41	23	0.086
**Diuretics, %**	52	39	0.200
**Oral anticoagulants, %**	22	16	0.427
**Antiplatelets, %**	48	26	0.011
**Oral hypoglycemic drugs, %**	11	12	0.922
**Insulin, %**	22	11	0.089
**Statins, %**	52	23	0.001
**Anti-SARS-CoV-2 vaccine, %**	75	62	0.190
**PaO_2/FiO_2 < 300, %**	45	51	0.619

**D-dimer, ng/mL**	1356 (744-3001)	1017 (604-1969)	0.435
**CRP, mg/dL**	4 (1.0-9.3)	4.4 (1.4-9.5)	0.782
**Procalcitonin, ng/mL**	0.2 (0.12-0.32)	0.13 (0.08-0.29)	0.275
**WBC, X 1000/ µL**	7.9 (5.9-12)	7.1 (5.1-10.3)	0.070
**Total cholesterol, mg/dL**	155 ± 30	152 ± 42	0.762
**LDL cholesterol, mg/dL**	90 ± 28	90 ±34	0.901
**HDL cholesterol, mg/dL**	43 ± 16	40 ± 15	0.370
**Triglycerides, mg/dL**	107 (90-162)	103 (76-137)	0.376
**CCI**	7 (5-9)	5 (3-7)	0.002
**SOFA score**	2 (1-4)	2 (1-4)	0.987
**PP score**	4 (3-6)	5 (3-6)	0.776

Table S5. Baseline characteristics of the study population according to the occurrence of arterial thrombotic events.

AF, atrial fibrillation; ACE, angiotensin converting enzyme; ASCVD, atherosclerotic cardiovascular disease; ARBs, angiotensin receptor blockers; BBs, beta blockers; BMI, body mass index; CCBs, calcium channel blockers; CCI, Charlson Comorbidity Index; CKD, chronic kidney disease; CRP, C-reactive protein; FiO2, fraction of inspired oxygen; HDL, high-density lipoprotein; LDL, low-density lipoprotein; Lp(a), lipoprotein(a); PaO2, partial pressure of oxygen; PP, Padua prediction; SOFA, Sequential Organ Failure Assessment; VTE, venous thromboembolism; WBC, white blood cells.

Values of variables are expressed as mean ± SD, median (interquartile range), or percentages.

	Venous thrombotic events	No venous thrombotic events	p
**Age, years**	80 ± 12	74 ± 17	0.009
**Male sex, %**	41	52	0.178
**BMI, Kg/m^2**	27 ± 4	26 ± 5	0.861
**Current smoking, %**	5	6	0.740
**Hypertension, %**	69	66	0.689
**Type 2 diabetes, %**	13	24	0.121
**CKD, %**	13	18	0.390
**ASCVD, %**	28	23	0.481
**AF, %**	15	20	0.518
**Previous VTE, %**	18	6	0.003
**ACE inhibitors, %**	26	27	0.849
**ARBs, %**	13	13	0.901
**BBs, %**	36	33	0.725
**CCBs, %**	18	24	0.691
**Diuretics, %**	46	40	0.422
**Oral anticoagulants, %**	5	17	0.045
**Antiplatelets, %**	33	26	0.352
**Oral hypoglycemic drugs, %**	8	12	0.419
**Insulin, %**	10	12	0.745
**Statins, %**	23	24	0.855
**Anti-SARS-CoV-2 vaccine, %**	61	62	0.913
**PaO_2/FiO_2 < 300, %**	64	49	0.093

**D-dimer, ng/mL**	1356 (663-4280)	1001 (604-1928)	0.046
**CRP, mg/dL**	6.2 (2.0-10.6)	4.3 (1.4-9.4)	0.212
**Procalcitonin, ng/mL**	0.15 (0.11-0.32)	0.13 (0.08-0.29)	0.379
**WBC, X 1000/ μL**	7.7 (4.1-12.2)	7.2 (5.1-10.3)	0.978
**Total cholesterol, mg/dL**	151 ± 43	153 ± 41	0.798
**LDL cholesterol, mg/dL**	89 ± 34	91 ± 33	0.811
**HDL cholesterol, mg/dL**	38 ±15	40 ±15	0.583
**Triglycerides, mg/dL**	121 (76-154)	102 (77-133)	0.268
**CCI**	4 (1-7)	5 (3-7)	0.304
**SOFA score**	3 (2-4)	2 (1-4)	0.131
**PP score**	5 (3-6)	6 (4-7)	0.007

Table S6. Baseline characteristics of the study population according to the occurrence of venous thrombotic events.

AF, atrial fibrillation; ACE, angiotensin converting enzyme; ASCVD, atherosclerotic cardiovascular disease; ARBs, angiotensin receptor blockers; BBs, beta blockers; BMI, body mass index; CCBs, calcium channel blockers; CCI, Charlson Comorbidity Index; CKD, chronic kidney disease; CRP, C-reactive protein; FiO2, fraction of inspired oxygen; HDL, high-density lipoprotein; LDL, low-density lipoprotein; Lp(a), lipoprotein(a); PaO2, partial pressure of oxygen; PP, Padua prediction; SOFA, Sequential Organ Failure Assessment; VTE, venous thromboembolism; WBC, white blood cells.

Values of variables are expressed as mean ± SD, median (interquartile range), or percentages.

	ICU admitted/died	No ICU admitted/ discharged alive	p
**Age, years**	83 ± 11	73 ± 17	< 0.001
**Male sex, %**	55	51	0.429
**BMI, Kg/m^2**	25 ± 3	26 ± 5	0.030
**Current smoking, %**	2	7	0.109
**Hypertension, %**	66	66	0.992
**Type 2 diabetes, %**	27	22	0.256
**CKD, %**	30	16	0.002
**ASCVD, %**	25	23	0.689
**AF, %**	28	18	0.036
**Previous VTE, %**	5	7	0.488
**ACE inhibitors, %**	18	28	0.048
**ARBs, %**	8	14	0.145
**BBs, %**	36	33	0.556
**CCBs, %**	25	23	0.819
**Diuretics, %**	53	38	0.009
**Oral anticoagulants, %**	18	16	0.710
**Antiplatelets, %**	29	27	0.662
**Oral hypoglycemic drugs, %**	13	11	0.634
**Insulin, %**	19	11	0.024
**Statins, %**	17	25	0.088
**Anti-SARS-CoV-2 vaccine, %**	57	63	0.330
**PaO_2/FiO_2 < 300, %**	70	47	< 0.001

**D-dimer, ng/mL**	1439 (856-3140)	989 (581-1945)	0.001
**CRP, mg/dL**	7.6 (3.4-11.6)	3.9 (1.3-9.2)	< 0.001
**Procalcitonin, ng/mL**	0.25 (0.15-0.72)	0.12 (0.07-0.25)	< 0.001
**WBC, X 1000/ µL**	7.4 (4.8-11.6)	7.2 (5.1-10.3)	0.610
**Total cholesterol, mg/dL**	145 ± 43	154 ± 41	0.125
**LDL cholesterol, mg/dL**	82 ± 35	92 ± 33	0.028
**HDL cholesterol, mg/dL**	35 ± 13	41 ± 16	0.003
**Triglycerides, mg/dL**	112 (91-166)	101 (75-130)	0.004
**CCI**	5 (4-7)	5 (3-7)	0.517
**SOFA score**	4 (3-6)	2 (1-4)	< 0.001
**PP score**	6 (3-7)	5 (3-6)	0.004

Table S7. Baseline characteristics of the study population according to ICU admission/in-hospital death.

AF, atrial fibrillation; ACE, angiotensin converting enzyme; ASCVD, atherosclerotic cardiovascular disease; ARBs, angiotensin receptor blockers; BBs, beta blockers; BMI, body mass index; CCBs, calcium channel blockers; CCI, Charlson Comorbidity Index; CKD, chronic kidney disease; CRP, C-reactive protein; FiO2, fraction of inspired oxygen; HDL, high-density lipoprotein; LDL, low-density lipoprotein; Lp(a), lipoprotein(a); PaO2, partial pressure of oxygen; PP, Padua prediction; SOFA, Sequential Organ Failure Assessment; VTE, venous thromboembolism; WBC, white blood cells.

Values of variables are expressed as mean ± SD, median (interquartile range), or percentages.

		Model 1	Model 2	Model 3	Model 4
**Dependent variable: thrombotic events**	**Lp(a), mg/dL**	1.002 (95%CI 0.988-1.015)	1.001 (95%CI 0.987-1.016)	*1.004 (95%CI 0.988-1.021)	°1.004 (95%CI 0.987-1.021)
	**Lp(a) > 13 mg/dL**	1.446 (95%CI 0.732-2.858)	1.308 (95%CI 0.654-2.616)	*1.219 (95%CI 0.571-2.602)	°1.204 (95%CI 0.551-2.632)
	**Lp(a) > 30 mg/dL**	1.103 (95%CI 0.478-2.545)	1.004 (95%CI 0.429-2.350)	*1.124 (95%CI 0.445-2.837)	°1.153 (95%CI 0.454-2.928)
	**Lp(a) > 50 mg/dL**	1.607 (95%CI 0.572-4.517)	1.615 (95%CI 0.562-4.645)	*1.608 (95%CI 0.496-5.211)	°1.642 (95%CI 0.506-5.328)
	**Lp(a) tertiles**	1.357 (95%CI 0.917-2.009)	1.298 (95%CI 0.871-1.936)	*1.313 (95%CI 0.843-2.046)	°1.291 (95%CI 0.825-2.021)
**Dependent variable: arterial thrombotic events**	**Lp(a), mg/dL**	1.009 (95%CI 0.993-1.026)	1.011 (95%CI 0.993-1.029)	**1.011 (95%CI 0.992-1.031)	°°1.012 (95%CI 0.992-1.032)
	**Lp(a) > 13 mg/dL**	2.050 (95%CI 0.655-6.416)	1.814 (95%CI 0.570-5.770)	**1.706 (95%CI 0.527-5.520)	°°1.768 (95%CI 0.490-6.384)
	**Lp(a) > 30 mg/dL**	1.873 (95%CI 0.557-6.302)	1.661 (95%CI 0.484-5.695)	**1.722 (95%CI 0.491-6.032)	°°2.041 (95%CI 0.556-7.489)
	**Lp(a) > 50 mg/dL**	3.204 (95%CI 0.827-12.406)	3.178 (95%CI 0.788-12.816)	**2.982 (95%CI 0.727-12.232)	°°3.561 (95%CI 0.839-15.114)
	**Lp(a) tertiles**	1.578 (95%CI 0.826-3.016)	1.492 (95%CI 0.771-2.889)	**1.466 (95%CI 0.747-2.875)	°°1.470 (95%CI 0.715-3.019)
**Dependent variable: venous thrombotic events**	**Lp(a), mg/dL**	0.994 (95%CI 0.974-1.014)	0.993 (95%CI 0.972-1.014)	***0.983 (95%CI 0.953-1.014)	°°°0.979 (95%CI 0.948-1.012)
	**Lp(a) > 13 mg/dL**	1.170 (95%CI 0.530-2.581)	1.092 (95%CI 0.490-2.432)	***0.820 (95%CI 0.284-2.370)	°°°0.785 (95%CI 0.261-2.359)
	**Lp(a) > 30 mg/dL**	0.689 95%CI 0.229-2.074)	0.642 (95%CI 0.212-1.947)	***0.583 (95%CI 0.151-2.242)	°°°0.522 (95%CI 0.129-2.106)
	**Lp(a) > 50 mg/dL**	0.775 (95%CI 0.174-3.460)	0.763 (95%CI 0.170-3.435)	***0.434 (95%CI 0.074-2.555)	°°°0.390 (95%CI 0.064-2.388)
	**Lp(a) tertiles**	1.223 (95%CI 0.776-1.927)	1.186 (95%CI 0.749-1.878)	***0.956 (95%CI 0.516-1.770)	°°°0.910 (95%CI 0.485-1.707)

Table S8. Odds ratios for the occurrence of thrombotic events according to Lp(a) levels in the subgroup of patients with severe COVID-19.

[1] Model 1: unadjusted. Model 2: adjusted for age and sex. Model 3: * adjusted for age, sex, ASCVD, previous VTE, , and in-hospital thromboembolism prophylaxis with LMWH; ** adjusted for age, sex, ASCVD, and in-hospital thromboembolism prophylaxis with LMWH; *** adjusted for age, sex, previous VTE, preadmission oral anticoagulants, PP score, D-dimer, and in-hospital thromboembolism prophylaxis with LMWH; ° adjusted for age, sex, ASCVD, previous VTE, anti-SARS-CoV-2 vaccine, and in-hospital thromboembolism prophylaxis with LMWH; °° adjusted for age, sex, ASCVD, anti-SARS-CoV-2 vaccine, and in-hospital thromboembolism prophylaxis with LMWH; °°° adjusted for age, sex, previous VTE, preadmission oral anticoagulants, anti-SARS-CoV-2 vaccine, PP score, D-dimer, and in-hospital thromboembolism prophylaxis with LMWH.

		Model 1	Model 2	Model 3	Model 4
**Dependent variable: ICU admission/in-hospital death**	**Lp(a), mg/dL**	1.003 (95%CI 0.993-1.013)	1.003 (95%CI 0.993-1.014)	1.009 (95%CI 0.997-1.022)	1.007 (95%CI 0.994-1.021)
	**Lp(a) > 13 mg/dL**	1.033 (95%CI 0.645-1.652)	1.014 (95%CI 0.633-1.625)	1.522 (95%CI 0.725-3.195)	1.242 (95%CI 0.593-2.603)
	**Lp(a) > 30 mg/dL**	1.101 (95%CI 0.598-2.025)	1.086 (95%CI 0.589-2.002)	1.009 (95%CI 0.416-2.451)	0.990 (95%CI 0.389-2.515)
	**Lp(a) > 50 mg/dL**	0.895 (95%CI 0.359-2.235)	0.900 (95%CI 0.360-2.247)	1.569 (95%CI 0.436-5.645)	1.367 (95%CI 0.346-5.406)
	**Lp(a) tertiles**	1.030 (95%CI 0.781-1.358)	1.015 (95%CI 0.768-1.342)	1.155 (95%CI 0.755-1.769)	1.090 (95%CI 0.715-1.662)

Table S9. Hazard ratios for ICU admission/in-hospital death according to Lp(a) levels in the subgroup of patients with severe COVID-19.

[1] Model 1: unadjusted. Model 2: adjusted for age and sex. Model 3: adjusted for age, sex, BMI, CKD, AF, preadmission ACE-inhibitors, preadmission diuretics, preadmission insulin, SOFA score, PP score, CRP, D-dimer, LDL cholesterol, HDL cholesterol, triglycerides, in-hospital corticosteroids, and in-hospital remdesivir. Model 4: adjusted for age, sex, BMI, CKD, AF, anti-SARS-CoV-2 vaccine, preadmission ACE-inhibitors, preadmission diuretics, preadmission insulin, SOFA score, PP score, CRP, D-dimer, LDL cholesterol, HDL cholesterol, triglycerides, in-hospital corticosteroids, and in-hospital remdesivir.

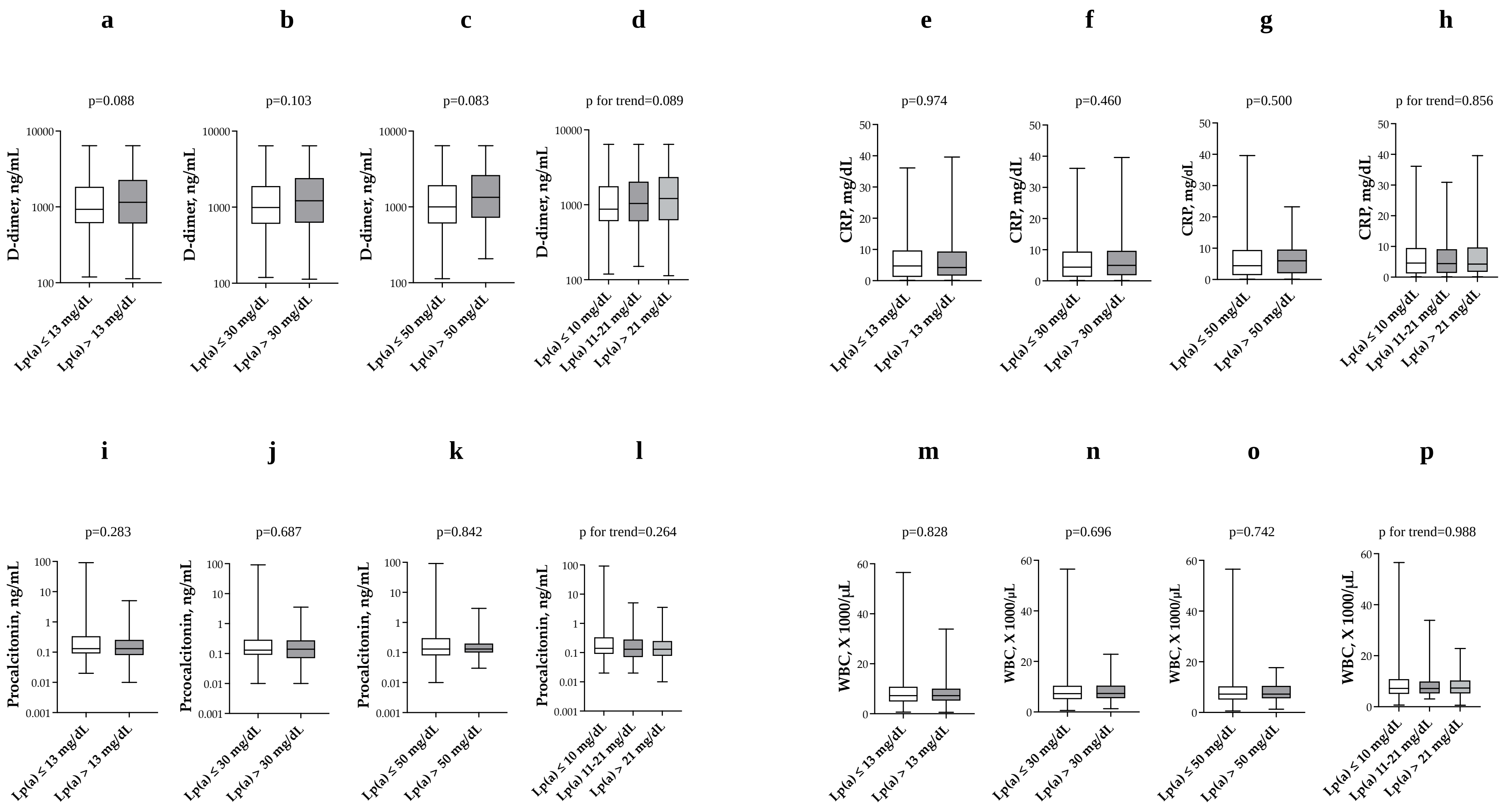

Figure S1. Biomarkers of thrombo-inflammation according to Lp(a) levels. (**a-d**) D-dimer according to Lp(a) levels. (**e-h**) CRP according to Lp(a) levels. (**i-l**) Procalcitonin according to Lp(a) levels. (**m-p**) WBC according to Lp(a) levels.

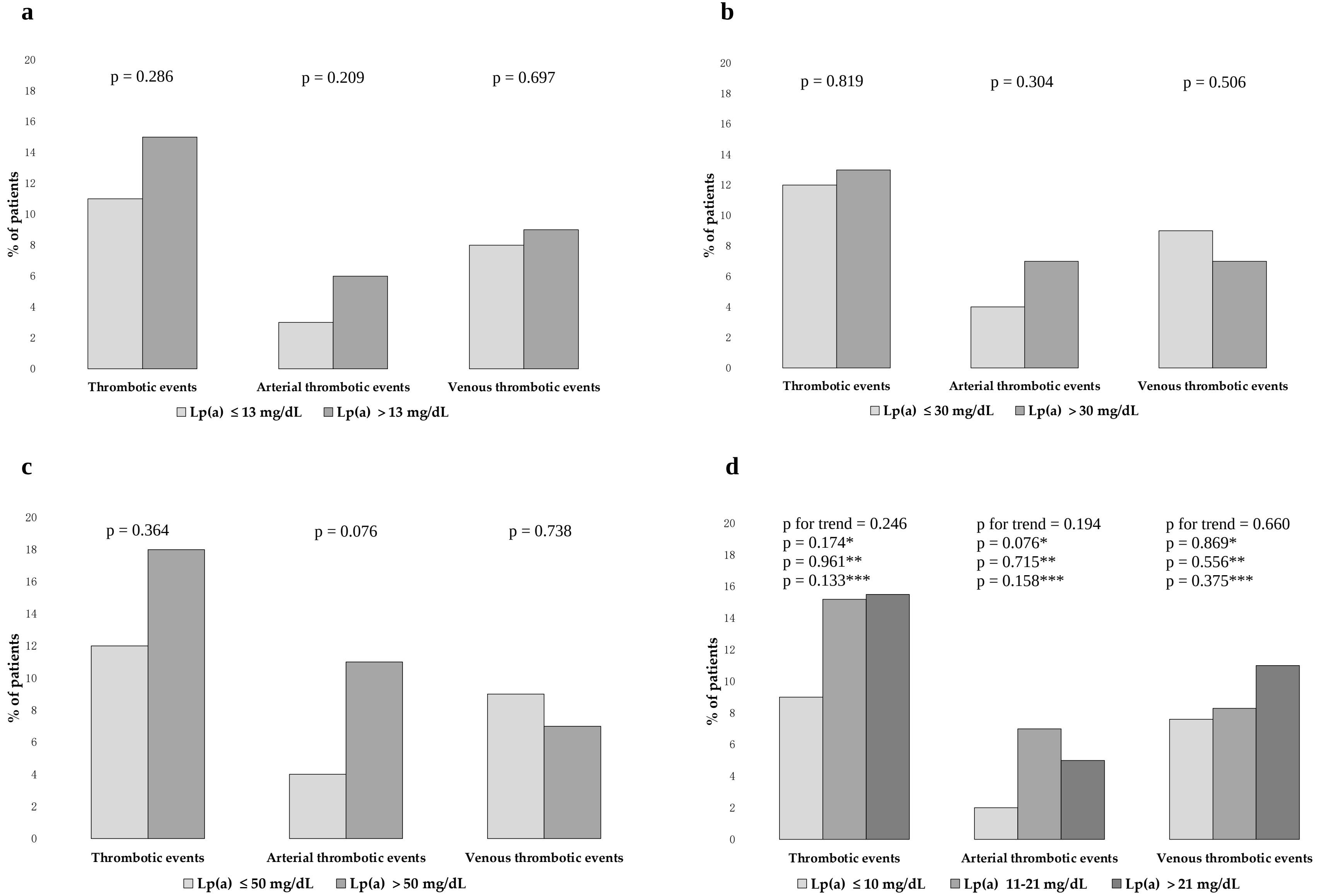

Figure S2. Rates of thrombotic events according to Lp(a) levels in the subgroup of patients with severe COVID-19. (**a**) Rates of thrombotic events (either arterial and venous combined or separated) according to Lp(a) ≤ *versus* > the median value. (**b**) Rates of thrombotic events (either

arterial and venous combined or separated) according to Lp(a) ≤ *versus* > 30 mg/dL. (**c**) Rates of thrombotic events (either arterial and venous combined or separated) according to Lp(a) ≤ *versus* > 50 mg/dL. (**d**) Rates of thrombotic events (either arterial and venous combined or separated) according to Lp(a) tertiles. * 1st *versus* 2nd tertile; ** 2nd *versus* 3rd tertile; ***1st *versus* 3rd tertile.

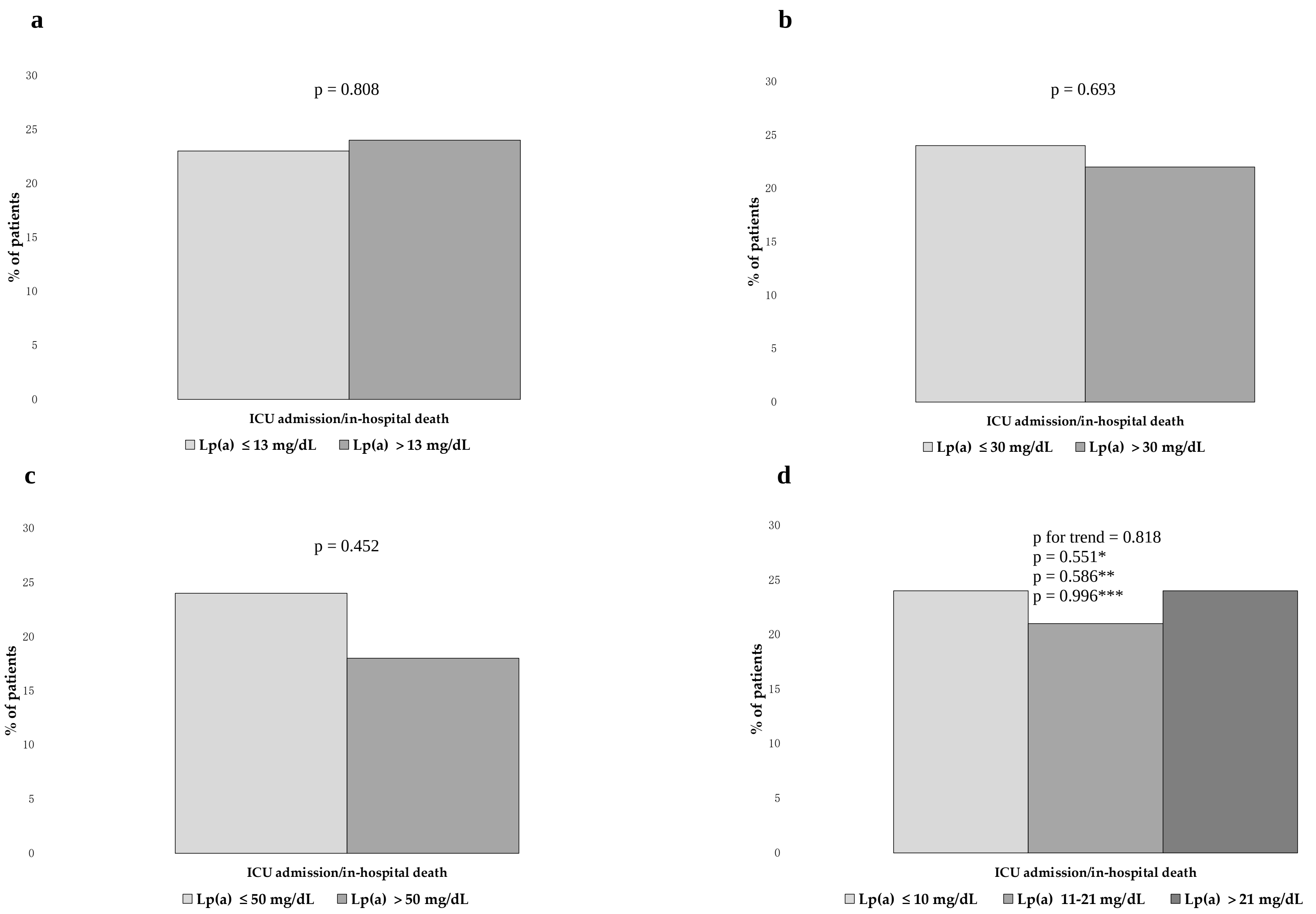

Figure S3: Rates of ICU admission/in-hospital death according to Lp(a) levels in the subgroup of patients with severe COVID-19. (**a**) Rates of ICU admission/in-hospital death according to Lp(a) ≤ versus > the median value. (**b**) Rates of ICU admission/in-hospital death according to Lp(a)

≤ versus > 30 mg/dL. (**c**) Rates of ICU admission/in-hospital death according to Lp(a) ≤ versus > 50 mg/dL. (**d**) Rates of ICU admission/in-hospital death according to Lp(a) tertiles. * 1st *versus* 2nd tertile; ** 2nd *versus* 3rd tertile; ***1st *versus* 3rd tertile.
